# Exploring magnetohydrodynamic voltage distributions in the human body: Preliminary results

**DOI:** 10.1371/journal.pone.0213235

**Published:** 2019-03-06

**Authors:** T. Stan Gregory, Jonathan R. Murrow, John N. Oshinski, Zion Tsz Ho Tse

**Affiliations:** 1 College of Engineering, University of Georgia, Athens, Georgia, United States of America; 2 AU/UGA Medical Partnership, University of Georgia, Athens, Georgia, United States of America; 3 Department of Radiology and Imaging Sciences, Emory University, Atlanta, Georgia, United States of America; 4 School of Electrical and Computer Engineering, University of Georgia, Athens, Georgia, United States of America; Policlinico S. Orsola-Malpighi, ITALY

## Abstract

**Background:**

The aim of this study was to noninvasively measure regional contributions of vasculature in the human body using magnetohydrodynamic voltages (V_MHD_) obtained from electrocardiogram (ECG) recordings performed inside MRI’s static magnetic field (B_0_). Integrating the regional V_MHD_ over the S_wave_-T_wave_ segment of the cardiac cycle (V_segment_) provides a non-invasive method for measuring regional blood volumes, which can be rapidly obtained during MRI without incurring additional cost.

**Methods:**

V_MHD_ was extracted from 12-lead ECG traces acquired during gradual introduction into a 3T MRI. Regional contributions were computed utilizing weights based on B_0_’s strength at specified distances from isocenter. V_segment_ mapping was performed in six subjects and validated against MR angiograms (MRA).

**Results:**

Fluctuations in V_segment_, which presented as positive trace deflections, were found to be associated with aortic-arch flow in the thoracic cavity, the main branches of the abdominal aorta, and the bifurcation of the common iliac artery. The largest fluctuation corresponded to the location where the aortic arch was approximately orthogonal to B_0_. The smallest fluctuations corresponded to areas of vasculature that were parallel to B_0_. Significant correlations (specifically, Spearman’s ranked correlation coefficients of 0.96 and 0.97 for abdominal and thoracic cavities, respectively) were found between the MRA and V_segment_ maps (p < 0.001).

**Conclusions:**

A novel non-invasive method to extract regional blood volumes from ECGs was developed and shown to be a rapid means to quantify peripheral and abdominal blood volumes.

## Introduction

This study aims to develop a noninvasive, MRI-compatible method for measuring regional blood volumes. Such a technology could be used to monitor risk factors for and track the progression of cardiovascular disease [1–6]. In addition, longitudinal usage might enable monitoring of wound healing [7]. This technique could be used during patient insertion into the MRI bore prior to the start of a new MRI study, providing additional information without any additional cost. In the future, the technique might also be applied using a static (neodymium) magnetic field source and a stand-alone hand-held device containing miniaturized ECG hardware, which would reduce the cost associated with utilizing an MRI scanner [8].

Our approach is inspired by past studies on utilizing electromagnetics to measure blood flow [9, 10]. The proposed technology is based on the magnetohydrodynamic (MHD) effect, a phenomenon in which the flow of charged particles in a direction perpendicular to a magnetic field creates an electric field in a direction which is mutually perpendicular to both the magnetic field and the flow [11]. A magnetic resonance imaging (MRI) scanner has a very strong static magnetic field ($\overset{⃑}{B_{0}}$), which at the center of the magnet is uniform and oriented along the shaft of the MRI bore, while at both ends of the bore it wraps around the exterior of the magnet, rapidly changing in strength and orientation. Induced MHD voltages (V_MHD_) are directly related to fluid velocity ($\overset{⃑}{u}$), magnetic flux density ($\overset{⃑}{B_{0}}$), and measurement electrode spacing (L) (Eq 1) [12–15].

$$V_{MHD}\;=\;\int_{0}^{L}(\overset{⃑}{u}\times \overset{⃑}{B_{0}}\;)∙\;\overset{⃑}{dL}$$ (1)

A well-known example of V_MHD_ arises when the human heart is placed at the center of the MRI scanner, where rapid ejection of blood from the left ventricle into the aortic arch induces a large V_MHD_, primarily because a sizeable length (5–10 cm) of the wide (~2 cm diameter) arch lies perpendicular to the magnetic field and the flow velocity is high (>100 cm/s). This V_MHD_ appears as a voltage overlaid on top of conventional electrocardiogram (ECG) traces measured inside the MRI bore, which peaks during cardiac systole [11, 12, 16–18]. Using a flow phantom placed inside an MRI, as well as a pump which provided pulsatile flow, V_MHD_ was reproduced in vitro. In addition, the correlation between V_MHD_ observed on conventional ECG traces and cardiac blood flow was demonstrated [19]. Current modeling approaches have been able to successfully simulate the induced V_MHD_ as a linear combination of the true ECG signal and a V_MHD_ term (Eq 1) with an additional scaling factor which was dependent on the measurement electrode selected [20]. Induced V_MHD_ was shown to increase with cross-sectional area and vessel diameter [20, 21]. As predicted, induced V_MHD_ increases as the MRI field strength increases [22, 23].

We hypothesized that monitoring the induced V_MHD_ during the incremental introduction of the human body into the MRI bore from regions completely outside the magnetic field afford an opportunity to assess regional vasculature contributions to the MHD effect, and to further utilize this data in order to assess blood volumes in various portions of the vasculature. A novel non-invasive method to extract regional blood volumes from ECGs was successfully developed and shown to be a rapid means to quantify peripheral and abdominal blood volumes.

## Methods

The study was conducted using a Signa HDx 3T MRI scanner (General Electric Healthcare, Waukesha, WI). A 12-lead ECG recording system, modified to be MR Conditional [24], was used to record the 12-lead ECGs of 6 volunteer subjects at 3T (Table 1).

**Table 1:** Body type and size of each volunteer subject.

	Gender	Age	Height (cm)	Weight (kg)
Subject #1	F	38	159	54.5
Subject #2	M	30	168	68.0
Subject #3	M	25	160	75.8
Subject #4	M	23	179	62.6
Subject #5	M	26	182	77.1
Subject #6	M	22	175	65.2

The human clinical trials in this study were approved by the University of Georgia Office of Research Institutional Review Board (IRB), and registered with the IRB (registration number: STUDY00003158) on 4/13/2016. Informed consent to participate in the study was obtained from all participants (or their parent or legal guardian in the case of children under 16). The consent forms are held by the authors and are available for review by the Editor-in-Chief. Written informed consent was obtained from all study volunteers for publication of their individual details and accompanying images in this manuscript (as per IRB procedures). The consent forms are held by the authors and are available for review by the Editor-in-Chief.

12-lead ECG patient monitoring was used as it is a clinical standard for cardiac monitoring that measures high-fidelity multi-channel bioelectric potentials at different physiological landmark positions of the thorax. As the study focused on the development of a new methodology for blood volume monitoring with a limited number of volunteer subjects, normal subjects without any prior history of cardiovascular diseases were chosen.

### Extraction of MHD voltage distributions

**Fig 1:**
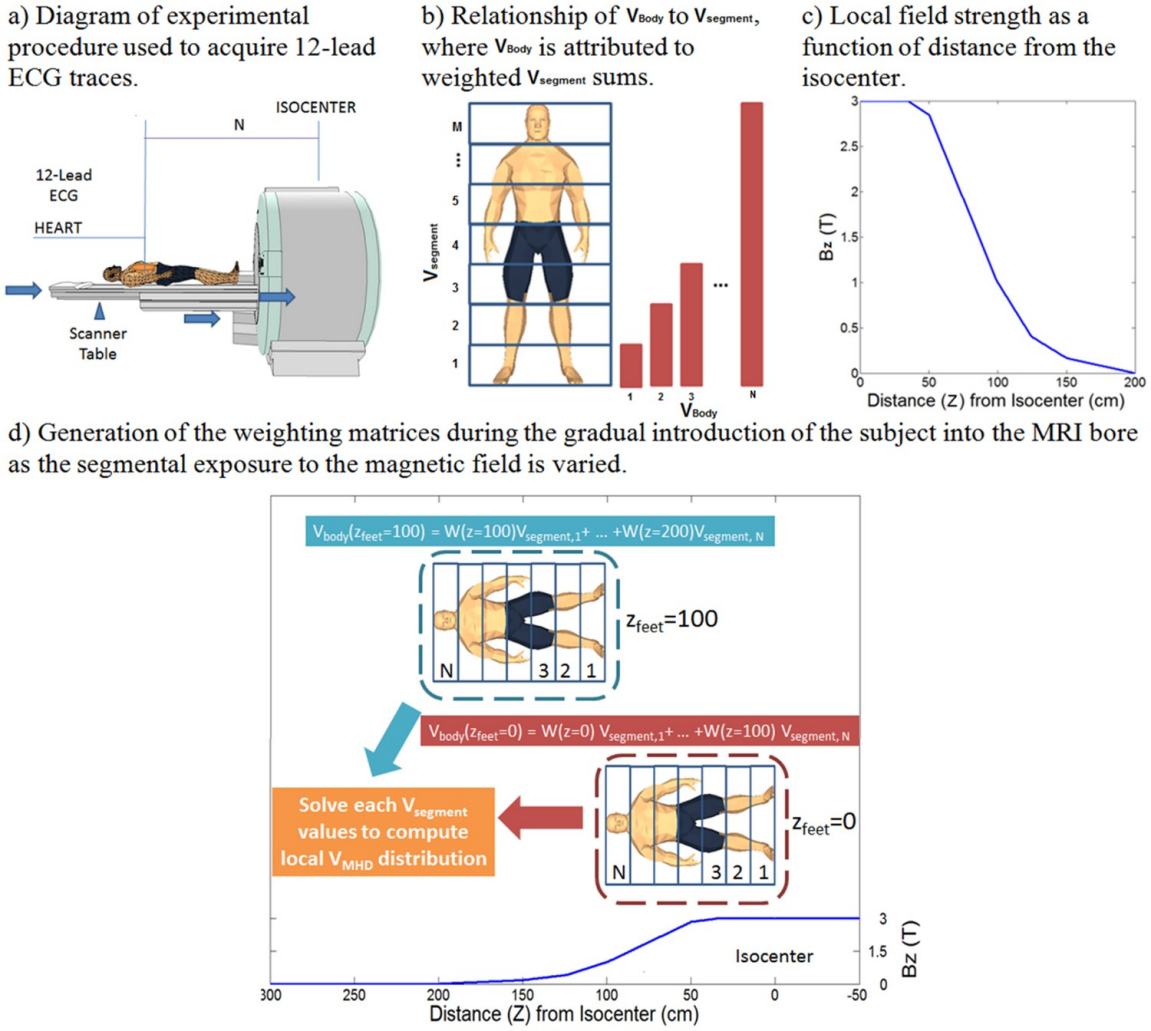
Recording of raw 12-lead ECG data for estimating regional contributions to the net V_MHD_ signal.

Baseline ECG recordings were obtained for each patient with the patient in the supine position outside the 5-gauss line of the MRI magnetic field. Subjects were placed supine on the MRI scanner table, in a feet-first orientation, with the table in a locked position and the cradle maximally extended out of the bore. The cradle was then advanced into the MRI, progressively bringing more parts of the human body into the MRI scanner’s fringe magnetic field, such that the induced V_MHD_ was observed to grow to beyond 5% of the ECG QRS complex. Introduction into the scanner bore was performed in 10-cm increments in five subjects (n = 5), terminating when the heart reached the scanner iso-center (Fig 1a). A higher-spatial-resolution dataset was recorded for an additional subject (n = 1) at 1-cm increments.

12-lead ECG recordings during 20-second breath holds were obtained at each position, characterized by its displacement from the MRI bore isocenter (displacement level). As this study focused on effects of the MRI magnetic field on 12-lead ECG recordings, data was obtained in the absence of MRI gradient pulses (i.e. without the occurrence of imaging). Twelve-lead ECG traces were converted into the VCG domain using an inverse Dower transform, providing a 3-component vector signal representation, [*VCG*_*X*_(*t*) *VCG*_*Y*_(*t*) *VCG*_*Z*_(*t*)] [25].

$$\left[\begin{array}{c} {VCG}_{X}\\ {VCG}_{Y}\\ {VCG}_{Z} \end{array}\right]\;=\;\left[\begin{array}{ccc} -0.172 & -0.074 & 0.122\\ 0.057 & -0.019 & -0.106\\ -0.229 & -0.310 & -0.246 \end{array}\;\begin{array}{ccc} 0.231 & 0.239 & 0.194\\ -0.022 & 0.041 & 0.048\\ -0.063 & 0.055 & 0.108 \end{array}\;\begin{array}{cc} 0.156 & -0.010\\ -0.227 & 0.887\\ 0.022 & 0.102 \end{array}\right]\;\left[\begin{array}{c} {ECG}_{I}\\ \begin{array}{c} {ECG}_{II}\\ {ECG}_{V1}\\ \begin{array}{c} {ECG}_{V2}\\ {ECG}_{V3} \end{array} \end{array}\\ \begin{array}{c} {ECG}_{V4}\\ {ECG}_{V5}\\ {ECG}_{V6} \end{array} \end{array}\right]$$ (2)

The VCG frame of reference provided a direct visualization of the cardiac electrical signal vectors in 3-dimensional (3D) space, where the magnitude and direction of signals were referenced to a dipole center. The assumption of the dipole center point of reference has been shown to hold true despite the moving depolarization and repolarization wave-fronts that occur in the atria and ventricles during a normal cardiac cycle [26, 27]. V_MHD_ in the VCG representation can visualize the magnitude and direction of V_MHD_ at different locations in the myocardium at different points in the cardiac cycle in 3D (8–10 cm during ventricular contraction) and allows direct comparison with MRI data acquired in the MRI coordinate planes. The VCG frame of reference is used in clinical diagnosis of cardiac arrhythmias originating from varying sources within the heart (such as the ventricular wall), which superimpose signals onto the acquired VCG [28, 29]. In the environment of the MRI scanner, induced V_MHD_ signals are similarly superimposed onto the VCG, and are the basis for translating to this frame of reference for analysis. In addition, VCG vector format simplifies the subsequent signal processing from handling the 12 ECG traces to the 3 VCG traces.

V_MHD_ vector extraction was performed through the subtraction of VCGs obtained within the magnetic field at each displacement level $\left(\overset{-}{ECG_{measured}(z)}\right)$ from a constant reference, VCGs obtained with the subject completely outside of the MRI magnetic field acquired at the beginning of the procedure ($\overset{⃑}{{ECG}_{reference}}$) (Eq 3). This serves to isolate the MHD signal contribution from the contribution of the true time-integrated ECG [24].

$$\overset{-}{V_{MHD}(z)}\;=\;\overset{-}{{ECG}_{measured}(z)}-\overset{⃑}{{ECG}_{reference}}$$ (3)

Time-integration of V_MHD_, corresponding to V_MHD_ induced by systolic blood flow, over the cardiac S-wave to T-wave (S-T) segment was performed at each displacement level, providing a metric proportional to blood volume through systolic integration of flow. This serves as a beat-to-beat estimate for the body’s contribution to the net recorded V_MHD_ (V_body_) in the three component directions [24] (Eq 4), the magnitude of which is then taken at each level. We defined V_body_ as the time integral of the recorded V_MHD_ magnitude over the duration of the S-T segment at each displacement level.

$$V_{body}\;=\;\int_{S}^{T}|\overset{⃑}{V_{MHD}(t)}|dt$$ (4)

The V_body_ metric can, therefore, be defined as the weighted summation of neighboring body segments (V_segment_), totaling the net induced MHD voltage recorded at each displacement level or V_body_ (Fig 1b). Since V_body_ is influenced by the varying magnetic field strengths as a function of subject displacement from the isocenter (Fig 1c), normalization or weighting terms must be included for comparison of the contribution of each V_segment_ (Eq 5). W(z) takes into account the spatial variation of the magnetic field (B_0_(z)) at various distances from magnet isocenter (Fig 1d) [30, 31].

This summation of each weighted V_segment_ term is performed over the entire height of the subject, forming a total of N discrete segments of equal length (t = 10 cm). Increases in N can result in higher spatial resolution for identifying the magnetohydrodynamic voltage distributions in the subject body.

$$W(z)\;=\;\frac{B_{0}(z)}{3T}$$ (5)

A linear decomposition was applied in order to resolve the weighted V_segment_ from the V_body_ measured in incremental distances from isocenter (Eqs 6–7). Each V_segment_ value was calculated at different body segments and scaled to be independent of applied magnetic field at each displacement level using *W*(z). V_segment_ values were calculated for each body segment of t cm, and repeatedly for N body regions (Fig 1d).

$$W(z)V_{segment}\;=\;V_{body}$$ (6)

$$\left[\begin{array}{ccccc} W\left(z=0\right) & W\left(z=t\right) & W\left(z=2t\right) & \ldots & W\left(z=Nt\right)\\ W\left(z=t\right) & W\left(z=2t\right) & W\left(z=3t\right) & \ldots & W\left(z=\left(N+1\right)t\right)\\ W\left(z=2t\right) & W\left(z=3t\right) & W\left(z=4t\right) & \ldots & W\left(z=\left(N+2\right)t\right)\\ \vdots & \vdots & \vdots & \ddots & \vdots \\ W\left(z=Nt\right) & W\left(z=\left(N+1\right)t\right) & W\left(z=\left(N+2\right)t\right) & \ldots & W\left(z=2Nt\right) \end{array}\right]\left[\begin{array}{c} V_{segment,\left(1\right)}\\ V_{segment,\left(2\right)}\\ V_{segment,\;\left(3\right)}\\ \vdots \\ V_{segment,N} \end{array}\right]=\left[\begin{array}{c} V_{body}\left(z_{feet}=0\right)\\ V_{body}\left(z_{feet}=t\right)\;\\ V_{body}\left(z_{feet}=2t\right)\\ \vdots \\ V_{body}\left(z_{feet}=Nt\right) \end{array}\right]$$ (7)

### Statistical analysis

For each subject, an MRI angiogram was obtained prior to V_MHD_ processing for validation of obtained V_segment_ traces; MR images were obtained using three-dimensional phase contrast balanced steady-state free precession (bSSFP) sequences without the injection of Gadolinium contrast media, TR: 7, TE: 4, Flip Angle: 20 degrees, 8 mm slice thickness. Image reconstruction and processing was performed using the OsiriX DICOM Viewer (Pixmeo, Bernex, Switzerland). Maximum intensity projections (MIPs) were performed across the patient angiogram at 8 mm intervals, and a thoraco-abdominal trace was obtained from the MRI angiogram. The correlation between V_MHD_ and the MRA curves was quantified using Spearman’s ranked correlation coefficient in both the abdominal and thoracic cavities.

## Results

**Fig 2:**
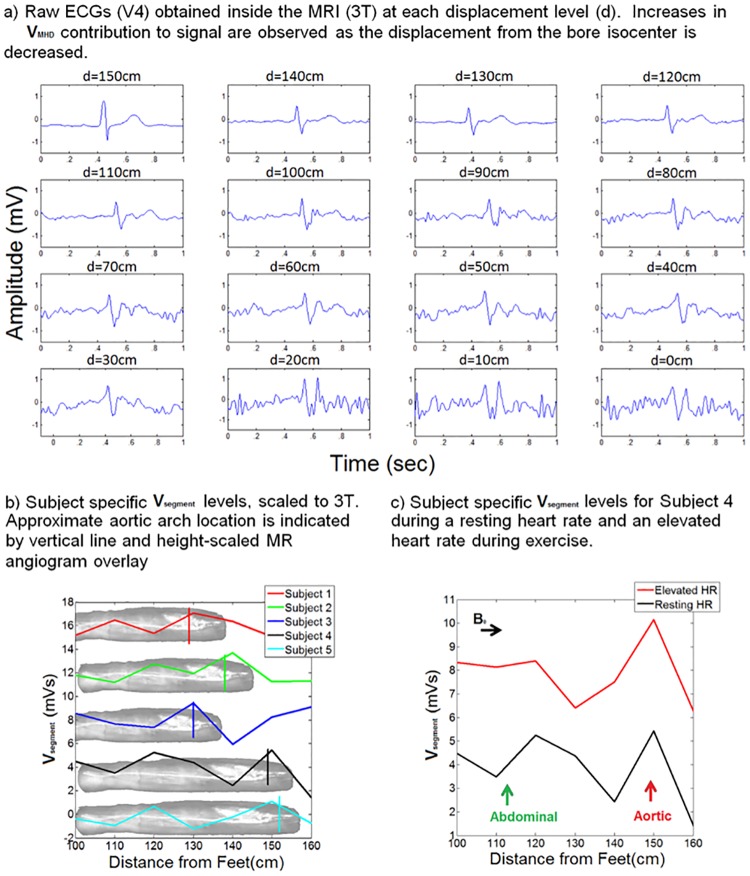
Derivation of subject-specific V_MHD_ based maps through the gradual introduction of the subject into the static magnetic field of the MRI.

V_segment_ was resolved in each subject (Fig 2b) and displayed as a function of the distance (displacement) from the subject’s feet or height.

### Extraction of MHD voltage distributions

V_segment_ varied over the course of the different body segments, with the major blood vessels providing for greater changes in V_segment_. Minimal V_segment_ intensity was found in vasculature oriented mainly parallel to the magnetic field, where the direction of blood flow aligned with B_0_ during the experimental procedure (Fig 2). This occurred for the abdominal aorta and the carotid artery in all subjects.

**Fig 3:**
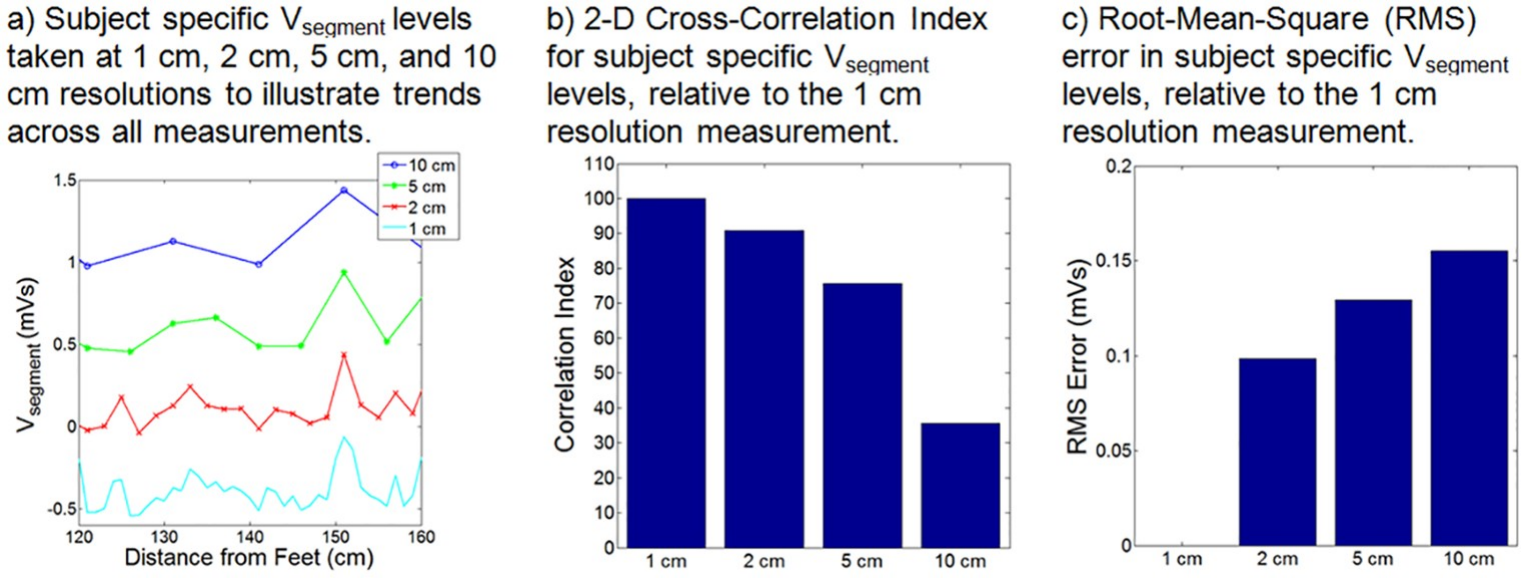
Subject specific V_segment_ levels obtained for a single subject at 1 cm, 2 cm, 5 cm, and 10 cm resolutions.

V_MHD,_ attributed to rapidly flowing blood in the aortic arch during early systole, was shown to dominate V_segment_ curves in all the subjects. A similar increase in V_MHD_ was observed due to blood volumes stored in the abdominal solid organs and fed by primary branches of the major vasculature (e.g. the abdominal aorta). The dataset obtained at the increased spatial resolution (1 cm) illustrated a similar trend as observed with the 10 cm resolution, as demonstrated by varying the spatial resolution of the acquired 1 cm high-resolution dataset to 2 cm, 5 cm, and 10 cm (Fig 3).

Fluctuations in V_segment_ (Fig 3a), which were primarily observed as positive trace deflections, were associated with aortic-arch flow within the thoracic cavity, the main branches of the abdominal aorta, such as the renal, splenic, and hepatic arteries, as well as the bifurcation of the common iliac artery. The largest fluctuation was observed at the 150 cm mark, corresponding to the location where the aortic arch was approximately angled orthogonal to B_0_.

A 50.6% increase in cardiac V_segment_ was observed in subject 4 (Fig 2c) when the heart rate was elevated from a resting heart rate of 82 bpm to 114 bpm during exercise, corresponding to an increased level of blood flow during exercise.

### Statistical analysis

**Fig 4:**
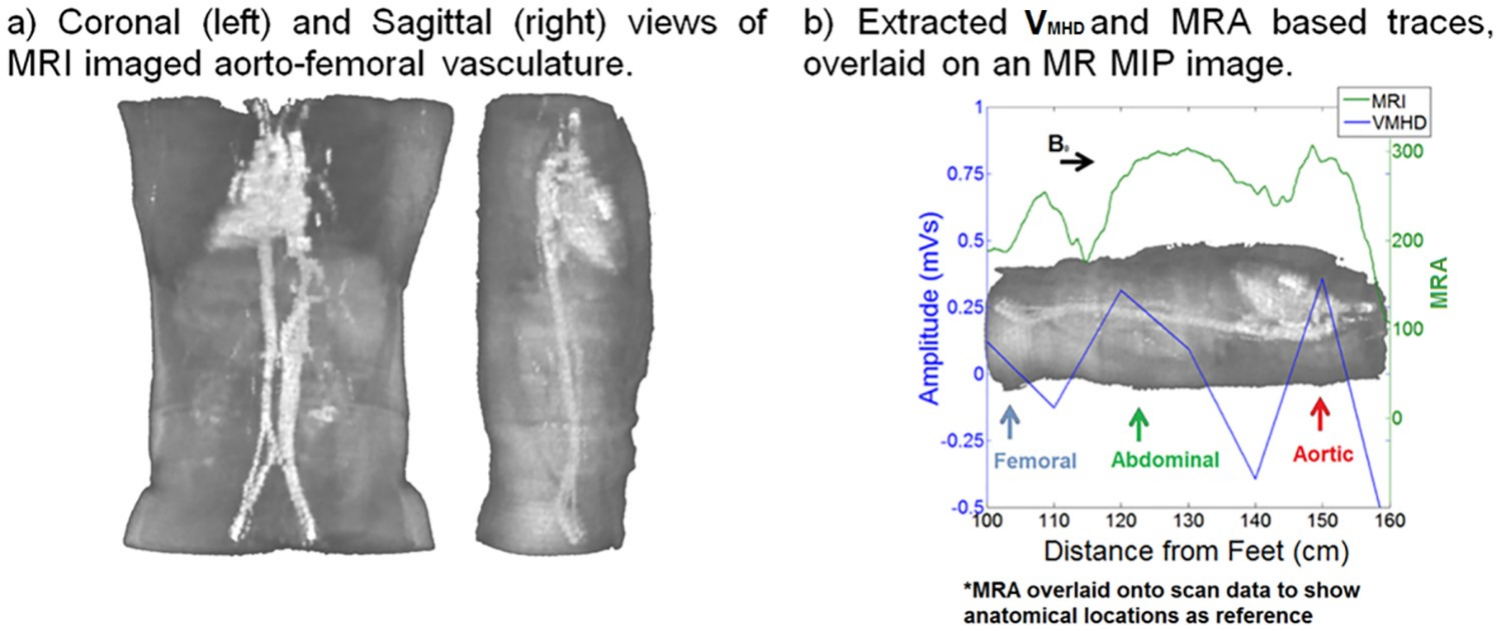
MRI validation of V_MHD_ based calculations using MR angiograms.

MR angiograms were obtained for each subject and subsequent trace extraction was performed. MRA traces were compared to the extracted V_MHD_ traces, as determined in (1), to assess method validity and overlaid onto MRA magnitude images (Fig 4).

A significant correlation, 0.96 and 0.97 for abdominal and thoracic cavities, respectively, was found between the MRA and V_MHD_ derived traces, with a p-value of <0.001 using Spearman’s ranked correlation coefficient, where a coefficient of 1 designates perfect correlation [32]. Comparison of V_MHD_- and MRA-based traces yielded parallel positive signal deflections in the subject’s superior thoracic cavity. These appeared at the level of the aortic arch, a known primary contributor to the net MHD voltage. Abdominal correlation is attributed to the large blood volume reserves contained in the abdominal solid organs, and the respective high blood-flow arterial branches out of the abdominal aorta. Renal, splenic, and hepatic branches of the abdominal aorta maintain large angles (~90 degrees) between the vasculature and the magnetic field direction, causing large increases in V_MHD_, which match that seen in the MRA projections. The MHD voltage was therefore demonstrated to reflect the net effect of blood flow occurring within individual body regions, as recorded through surface ECGs, in a similar manner to that obtained through traditional MRA procedures.

## Discussion

Subject specific V_segment_ levels were extracted and shown to correlate with subject anthropometry. A high-resolution dataset was acquired (1 cm) and shown to correlate well with the standard 10 cm resolution measurements.

MRA-derived measurements were shown to agree well with V_MHD_-based experimental data, excluding specific regions where the V_MHD_-derived maps displayed lower blood flow, because the direction of blood flow in these regions was mainly parallel to the magnetic field.

The development of a rapid non-invasive measurement of patient blood-volume levels would allow physicians to more accurately detect changes in flow and blood volume across a wider population, including in patients with peripheral arterial disease or congestive heart failure. It is expected that the V_MHD_ maps would detect restricted flow cases.

As is true for any MRI procedure, patients with medical implants not labeled as MR conditional in the 3T field, would need to be excluded from this procedure. Additional patient safety concerns related to RF fields and static/gradient magnetic fields were previously studied and found to be limited to induced nausea during gross patient movements, which is considered as non-significant risk to patients at 3T [30, 31]. Moreover, in order for the proposed method to be accepted for use with patients with pathologies, such as those caused by cardiovascular diseases, it is important to understand the effects of magnetic fields on abnormal vasculature. For example, blood flow through stenosed arteries [33], irregular multi-stenosed arteries [34], and aneurysmal geometry [35] has been found to react to magnetic fields differently than blood flow through healthy vasculature, sometimes with complex responses.

Further studies to increase method accuracy and reduce the required acquisition time must be performed to integrate this technology into existing MR scanner platforms or to develop a standalone device for measuring blood volumes.

### Limitations

This study is a feasibility study to explore the use of the MHD effect as a novel vascular metric. The limited population size in this study necessitates that the method be further validated in a larger population, including in patients diagnosed with cardiovascular diseases. The present work presents a proof of concept study for examining regional body contributions to the net recorded V_MHD_.

The V_MHD_ maps may, however, also be influenced by other sources, such as large chest dimensions and increased layers of subcutaneous fat. Further studies must be performed to fully understand the role of variations in tissue electrical conductivity on the proposed methodology. Furthermore, the VCG frame of reference normally models the recorded V_MHD_ as originating in a single dipole source within the heart, whereas this study seeks to find contributions to the net V_MHD_ from several sources located far from the heart. In the case of V_MHD_ sources located near the SA node, which have comparable displacements of the detecting electrodes from the cardiac dipole source, such as the MHD voltage originating from the aortic arch, we have shown that this assumption is valid. Larger source displacements from the heart, such as those studied here, may require modifying the Dower Transform, and generating subject-specific coefficients. Subject-specific coefficients have been shown to provide increased accuracy relative to the generic coefficients [36]. We limited this study to the thoraco-abdominal cavity in order to reduce this variability. However, the merits of generating subject-specific coefficients should be further explored, since they may increase the accuracy of the blood-volume maps.

The presented method demonstrated the contributions of vasculature in the human body to the net MHD voltage, but did not provide a quantitative transfer-function between the resolved V_segments_ and the true blood volume distributions. To completely develop this transfer function, a quantitative analysis is required to understand the relationship between the MHD voltages, the size and velocity of the vessels of interest, and the attenuation of electrical signal as they traverse through different tissue layers along its path from the MHD source to the surface-electrode detectors.

In addition, the current method is limited to V_MHD_ contributions from vasculature lying primarily perpendicular to the MRI’s magnetic field direction. This is because when the blood flow is perpendicular to the magnetic field, the magnetic force on the blood is maximum. In contrast, when the blood flow is parallel to the magnetic field, there is no magnetic force on the blood, so the movement of the blood is not affected. Hence, V_MHD_ contributions from vasculature lying primarily parallel to the MRI’s magnetic field direction are minimal. Therefore, the proposed method of using MHD for flow estimation is most sensitive to blood flow perpendicular to the magnetic field. In order to add contributions from vasculature that lie in other orientations, magnetic fields oriented in other directions could be added, which might be possible to perform by adding bucking coils outside the MRI bore.

Although the main field of MRI scanner is in the z direction, there are secondary magnetic fields in the x and y direction near the gantry of the MRI scanner, which can produce small undesirable MHD voltages and therefore affect the measurement accuracy. However, the undesirable MHD voltages are far away from the measurement electrodes, so the sensor will not pick up very much noise. In future studies we will explore a way to consider the x and y magnetic field in our computation for blood flow measurements.

The current method is based on Dower transform to convert ECG into VCG domain. The Dower transform is a well-known method for ECG conversion, but it makes assumption about the size, shape, electrical properties, and signal source of the parcel, which affects the accuracy of the calculation. Future work will include multi-electrode measurements across the whole torso in order to produce more accurate MHD measurements.

Another limitation is that patients tend to have bad ECG measurements while exercising.

Measurements during heavy exercise tend to have severe noise interference and baseline rendering which can affect measurement accuracy.

### Future Work

Further work to advance the utility of the study includes the development of a standalone device that is based on a portable magnetic field source and miniaturized ECG hardware, allowing for metric quantification in the absence of the MRI magnetic field. The methods presented will also be further validated in a larger subject population that contains both healthy subjects and patients with vascular diseases, such as peripheral arterial disease or congestive heart failure. Future work must be performed to map higher resolution V_MHD_-derived features to MRI scan data, and to relate the MHD spatial decomposition to flow distributions in the body.

## Conclusions

In this study, V_MHD_ signals were shown to correlate well with regional blood volumes, illustrating trends comparable to those from standard imaging methodologies. Although the scope of this study was limited, the preliminary results supported the proof of concept. Therefore, future studies are warranted to rigorously investigate the proposed method. Moreover, future studies should consider the integration of this method into a portable device to improve clinical workflows.
